# The impact of public art education on college students’ mental health literacy

**DOI:** 10.3389/fpubh.2024.1427016

**Published:** 2024-08-26

**Authors:** Shengyu Zhang, Lege Zhao

**Affiliations:** ^1^School of Advertising, Communication University of China, Beijing, China; ^2^College of Humanities and Communication, Dongbei University of Finance and Economics, Dalian, China

**Keywords:** public art education, college students, mental health literacy, psychological intervention, educational outcomes

## Abstract

**Introduction:**

This study aims to explore the impact of public art education on the mental health literacy of College Students.

**Methods:**

Conducted over a four-month period, the intervention involved freshmen from a Chinese college, comparing 1,334 students in the experimental group with 1,139 in the control group. Data were collected through a self-developed questionnaire and a standardized mental health literacy scale before and after the intervention.

**Results:**

Results showed that the experimental group’s overall mental health literacy score significantly increased from 64.051 pre-intervention to 79.260 post-intervention, while the control group showed no significant changes during the same period. The experimental group demonstrated significant improvements in their ability to identify psychological disorders, belief in receiving professional help, attitudes towards seeking appropriate help, and knowledge in seeking mental health information. Furthermore, the frequency of classroom interaction was positively correlated with improvements in mental health literacy (regression coefficient = 2.261***), highlighting the critical role of active participation in public art education settings.

**Conclusion:**

This study underscores the importance and effectiveness of implementing public art education in higher education and provides empirical support for future educational policies and practices.

## 1 Introduction

Entering college is a critical transition for young students, marked by challenges like academic pressures, social relationships, and self-identity exploration, which can lead to psychological issues such as anxiety and depression (1). Enhancing mental health literacy among college freshmen is therefore essential (2, 3). Mental health literacy, as defined by Jorm et al., involves not only basic knowledge of mental health but also the skills to identify, manage, and seek professional help for psychological issues (4). Research indicates that students with higher mental health literacy manage stress more effectively, exhibiting improved learning and social skills, which positively impacts their academic and personal development (5–9). Universities, as primary environments for young students, are obliged to boost mental health literacy through educational interventions (10), making the exploration and implementation of effective mental health education programs crucial, especially those that integrate public art education.

Public art courses play a unique and crucial role in enhancing College Students’ mental health literacy (11, 12). Public art education, by providing opportunities for emotional expression and release, creative thinking and self-awareness, as well as social interaction and teamwork, not only helps students effectively manage their emotions and relieve stress, but also enhances their social skills and team spirit. This, in turn, positively impacts their mental health literacy. These activities not only help students manage emotions and stress but also improve their social skills and team spirit, thereby boosting their mental health literacy. According to Csikszentmihalyi’s “flow” theory, engaging deeply in artistic creation can lead to a pleasurable state that significantly alleviates psychological stress and enhances mental health (13, 14). Art education offers a platform for students to express emotions and experiences through various forms, facilitating stress relief and promoting emotional management and self-awareness (15). The collective nature of art activities also strengthens students’ social abilities and teamwork, recognized as key in mitigating mental health problems (16, 17). Furthermore, the multimodal interaction in art education supports Vygotsky’s sociocultural theory, which emphasizes the role of social interaction and cultural tools in developing psychological functions (18, 19). Therefore, integrating art into mental health education not only improves students’ mental health literacy but also enhances their self-expression and creative problem-solving abilities while boosting their social adaptability.

However, despite the theoretical benefits of art education on mental health, further research and exploration are needed on how to implement it effectively in practice and the sustainability and depth of its effects. Thus, this study employs both quantitative and qualitative research methods, conducting an empirical study with freshmen at a Chinese college. We will reveal the specific effects of public art courses on students’ mental health literacy.

## 2 Literature review

### 2.1 Research on college students’ mental health literacy

The concept of mental health literacy was first introduced by Jorm et al. in 1997, defined as “a set of knowledge and beliefs that help individuals identify, cope with, and prevent psychological disorders” (4). In 2012, Jorm further developed the concept of mental health literacy, adding the content of helping others, summarized into five dimensions: understanding the prevention of mental disorders, recognizing the onset and progression of mental disorders, understanding help-seeking and treatment methods, knowledge of self-help strategies, and the skills when others are affected by mental disorders (20). Based on this theory, O’Connor M and others further categorized mental health literacy into three dimensions: “recognition of mental disorders, ““knowledge associated with mental disorders, “and “attitudes towards mental disorders” (8, 21). As research deepened, the concept of mental health literacy gradually expanded from simple knowledge dissemination to more comprehensive strategies for managing and promoting mental health. OConnor and Casey (21) and Liu (22) proposed that College Students’ mental health literacy includes the ability to identify psychological disorders, belief in receiving professional help, promoting appropriate help-seeking attitudes, and knowledge of seeking mental health information (22). Globally, mental health literacy has become a significant issue in public health, encompassing not only basic knowledge and identification skills of mental disorders but also extending to effective help-seeking, understanding of treatment methods, and application of self-help strategies. Modern mental health literacy theory also emphasizes the importance of reducing prejudice and discrimination against mental illnesses (23, 24), which plays a crucial role in improving the acceptance and effectiveness of mental health services. Furthermore, Kutcher and others have extended research on mental health literacy into the educational setting, especially among adolescents and College Students, emphasizing improving mental health literacy through educational interventions to prevent mental health issues and promote early intervention (7).

College Students, as a key group in mental health literacy research, have their psychological health status directly affecting their learning outcomes and future career development. Students with higher mental health literacy can identify and address psychological issues earlier (25, 26), for instance, by increasing recognition of mental disorders and reducing the stigma associated with seeking help, which aids in obtaining more timely and effective treatment (27). As research on College Students’ mental health literacy has progressed, it has covered evaluations, influencing factors, intervention effects, and long-term impacts. These studies not only help us understand the behavioral patterns of College Students facing psychological pressures and challenges but also provide a scientific basis for strategies to enhance mental health. In terms of evaluating mental health literacy, existing studies typically assess it through multidimensional scales covering knowledge of mental health, ability to recognize mental disorders, self-help strategies, help-seeking behavior, and attitudes towards mental disorders (4, 20, 21, 28). Additionally, researchers have developed mental health literacy assessment tools suitable for Chinese College Students, which often consider cultural differences and are more applicable to the actual conditions of domestic students (29). Regarding influencing factors, College Students’ mental health literacy is affected by various factors such as personal background (gender, grade, major), psychological traits (self-esteem, resilience), and environmental factors (family support, campus culture) (30, 31). Gender differences are an important influencing factor, as studies have shown that female College Students are generally more sensitive to recognizing mental disorders but may hesitate in help-seeking behaviors due to societal role expectations (32). The field of study is also an important factor; students in arts and medical fields tend to have more understanding and knowledge of mental health issues due to their curriculum. Regarding intervention measures, scholars have attempted to enhance College Students’ mental health literacy through various educational and support programs, including workshops, lectures, interactive courses, and online self-help programs (33). Research indicates that structured psychological education courses can effectively enhance College Students’ knowledge of mental health and coping skills, reducing the incidence of psychological issues (34). Moreover, long-term follow-up studies help scholars understand the enduring effects of mental health literacy interventions (35). These studies show that systematic mental health education can form a long-term mechanism among College Students, not only enhancing their mental health literacy but also improving their overall learning and life quality. Additionally, these interventions have been found to positively impact students’ career development, social skills, and societal adaptability.

However, despite numerous studies focusing on the positive impacts of mental health literacy on College Students, relatively few have explored the specific contributions of public art education courses to enhancing mental health literacy. Public art education, through offering diverse artistic experiences such as music, painting, and drama, not only fosters students’ emotional expression and creative thinking but also provides new pathways and methods for mental health education. The unique value of public art education lies in its ability to help students establish positive self-identity through artistic creation activities, thereby potentially preventing and alleviating psychological stress to some extent. Thus, more attention is needed on the potential role of public art education in enhancing College Students’ mental health literacy, exploring its applicative value in higher education and its specific impacts on students’ mental health.

### 2.2 Research on public art education

Public art education is a branch of the arts that focuses on social interaction and cultural participation, emphasizing the educational and social functions of art activities in public spaces (36). The core of this education model is to enhance the public’s aesthetic ability, cultural literacy, and sense of social responsibility through art. Public art education extends beyond the creation and performance of art to include how art serves as a tool to influence and improve the social environment (37, 38). It transcends traditional artistic venues, integrating art into everyday public life, inspired by John Dewey’s philosophy that education should be a meaningful, living experience, with art as an ideal medium (39, 40). The theoretical foundation of public art education also includes the notion of the democratization of culture, which asserts that everyone should have the right to access and enjoy cultural resources (41). Art educators promote this by bringing art into communities and schools, facilitating educational and social reforms (42, 43) and using art for social critique (44). Art is not only an object of aesthetics but also a tool for reflecting and critiquing social phenomena (45). Within this framework, art education is seen as a platform for fostering critical thinking and social awareness, using artworks to address and solve social issues such as inequality, discrimination, and environmental degradation.

Globally, public art education has become an important means of cultivating students’ comprehensive qualities and innovative abilities in higher education (46, 47). Numerous educational institutions have introduced a wealth of public art courses and projects aimed at enhancing students’ aesthetic appreciation, creativity, and social responsibility. However, while well-supported in the USA and Europe with diverse (48), technologically integrated teaching methods (49), it faces challenges in developing countries like China, where it is not compulsory, and resources are limited (50). Firstly, public art courses are not yet compulsory in many universities (43); secondly, compared to traditional academic courses, investment in and resources for art education are often limited (51). Moreover, the content and methods of education are sometimes conservative, focusing more on theoretical teaching than on practice and creation, lacking alignment with international art education standards (52). Furthermore, while many universities have recognized the importance of public art education and have begun to integrate more interdisciplinary teaching and cooperative projects, the professional training of teachers and student engagement still need enhancement. The teaching staff needs further professional development in artistic skills and educational methods to meet the demands of public art education. Student participation should not be limited to classroom learning but should also extend to social practices outside the campus, deepening their understanding and application of public art through practical projects.

Public art education significantly boosts college students’ mental health by fostering creativity, emotional expression, and social skills, which are essential for managing stress and enhancing self-esteem (53–55). Moreover, public art projects often require teamwork, which not only enhances students’ social skills but also helps build supportive social networks, a key factor in mental health. Art also promotes cultural resonance and a sense of belonging, making students feel they are part of the community, which is vital for preventing feelings of loneliness and isolation (56). More importantly, art activities provide a positive way to cope with life’s challenges, helping students maintain psychological resilience in the face of stress and uncertainty. Despite this, scholars have not yet fully explored the empirical impact of public art education on College Students’ mental health. While current theoretical research suggests its potential positive effects, further empirical studies are necessary to validate these theoretical assumptions and provide concrete evidence for universities to improve their art education programs. Thus, exploring the empirical impact of public art education on College Students’ mental health literacy is not only likely to enrich existing academic research but also guide higher education institutions in implementing mental health promotion strategies.

## 3 Research subjects and methods

### 3.1 Research subjects

This study will focus on the freshman class of 2023 at a Chinese university to explore the impact of public art education on their mental health literacy. Due to limitations in teacher availability and course planning, public art education courses were only offered to freshmen in certain colleges from September to December 2023, while freshmen in other colleges will take the same courses from March to June 2024. Therefore, students from the colleges that participated from September to December 2023 were assigned to the experimental group, while those who did not receive the courses were assigned to the control group. This arrangement ensures that during the same period, only the experimental group received the intervention, and the control group was not influenced by any art education intervention.

The intervention was developed by our research team, which includes faculty members from the Communication University of China and Dongbei University of Finance and Economics. The intervention activities in this study are based on the integration theory of art education and mental health. The course content includes various forms of art, such as painting, drama performance, and music creation, with each session centered around themes of mental health. Each class not only features art creation activities but also integrates theoretical knowledge of mental health and interactive feedback sessions. These course contents are closely linked to the primary outcomes measured by the Mental Health Literacy Scale (MHLS) to ensure consistency between the intervention’s objectives and measurement indicators. These activities aim to promote emotional regulation and self-awareness among students through artistic expression, effectively supporting their mental health needs. In the development process, we referenced existing literature and preliminary experimental studies conducted with similar populations to determine the most suitable types of activities and implementation methods for this study’s objectives. The specific course framework is shown in Table 1. The intervention program has been previously trialed with other grade groups at the university to explore the effect of art activities on enhancing mental health literacy. In this study, the intervention lasted 4 months, including 16 sessions, each approximately 90 min long. The content of the courses included guidance on artistic creation, discussions on mental health topics, and interactive feedback sessions. This structured and continuous intervention aims to deeply understand the impact of art education on students’ psychological states.

**Table 1:** Course Framework.

**Chapter**	**Chapter Title**	**Course Content Summary**	**Class Hours**
1	Introduction to Mental Health and Art	Introduction to basic concepts of mental health and its relationship with art.	3 sessions
		Session 1: Fundamentals of Mental Health	
		Session 2: Art as a Medium of Emotional Expression	
		Session 3: Application of Art in Psychotherapy	
2	Drawing and Emotional Expression	Identifying Emotions through Drawing	3 sessions
		Session 1: Drawing and Emotional Expression	
		Session 2: Creating Emotional Themed Drawings	
		Session 3: Analyzing Artwork and Emotional Reflection	
3	Theater and Self-Awareness	Role-Playing in Theater and Psychological Exploration	3 sessions
		Session 1: Self-Expression and Communication Skills	
		Session 2: Teamwork and Interaction in Theater	
		Session 3: Integration of Personal Experiences in Theater Activities	
4	Music Creation and Emotional Regulation	The Relationship between Music and Emotion	3 sessions
		Session 1: Music Creation Exercises	
		Session 2: Emotional Analysis and Sharing of Music Pieces	
		Session 3: Exploration of Music Therapy Techniques	
5	Visual Arts and Cultural Reflection	Cultural Symbols and Psychological Interpretation in Visual Arts	3 sessions
		Session 1: Exploring Mental Health in Cultural Contexts	
		Session 2: Creation and Exhibition of Visual Artworks	
		Session 3: Group Critique and Cultural Analysis of Artworks	
6	Integrated Art Creation and Mental Health	Multimedia Art Creation	3 sessions
		Session 1: Analyzing the Impact of Art on Mental Health Education	
		Session 2: Course Summary and Future Application	
		Session 3: Reflection and Feedback on Course Experiences	

According to the school’s teaching and curriculum requirements, each class involved one main instructor and two professionally trained teaching assistants. Thus, during the intervention, each class was guaranteed to be led by the main instructor, with two assistants to help conduct the course. There were no instances where the main instructor could not attend, as per school regulations class absence by a teacher is considered a teaching incident. These assistants and counselors not only have an artistic background but also understand mental health theory, ensuring they can smoothly guide students in artistic creation and provide necessary psychological support in the absence of a teacher. During class periods, according to school curriculum design requirements, students were informed at the beginning of the course about the schedule, objectives, and assessment requirements. Students were reminded that their classroom performance, including attendance and interaction during class, would count toward their continuous assessment. The assistants were responsible for recording students’ classroom performances and interactions with the facilitators. Additionally, all intervention activities were supervised by the school’s mental health center to ensure the quality and safety of the intervention.

In determining the sample size, the study used the common sample size calculation formula: *n* = (Z_α/2_/δ)^2^*p*(1 − p), where *Z*_α/2_ is the *Z*-value corresponding to the confidence level, calculated at 95% confidence level (α = 0.05), with a corresponding *Z*-value of 1.96. Considering potential data fluctuations and response biases that might occur in practical research, an error margin δ of 0.02 was set. Based on the average prevalence rate of depression symptoms among Chinese College Students over the past 10 years at 31.4% (57), the *p*-value was set at 0.32 to reflect the proportion of the population with this characteristic. The choice of this indicator is primarily based on the following reasons: (1) Representativeness of the prevalence rate: By referring to data from the past 10 years, we are able to obtain a stable and widely accepted prevalence rate of depression, which helps ensure the representativeness and breadth of our study sample. Mental health issues among Chinese college students, particularly depression, have become a significant public health concern. Thus, selecting the average prevalence rate for this specific population provides a practical and relevant foundation for the study. (2) Statistical power: Using the actual prevalence rate to calculate the sample size helps ensure that the study has sufficient statistical power, i.e., the ability to detect the effects of experimental interventions. If the sample size is too small, important effects may not be detectable; if too large, it may be uneconomical and unnecessary. (3) Empirical evidence: Using data from the past decade not only provides empirical evidence on disease trends but also reflects epidemiological changes that may occur over different time periods, making the research results more relevant to the present and explanatory. Using these parameters, the theoretical sample size calculated was approximately 2090 students. However, to compensate for potential non-responses and sample attrition during data collection, an attrition rate of 20% was anticipated. This means that to ensure the statistical power of the study, the final number of surveys distributed needed to exceed 2,508 students. This sample size not only enhances the representativeness and reliability of the study results but also ensures sufficient statistical power in data analysis to identify any actual impacts of public art education on the mental health literacy of college freshmen. Through such rigorous sample size settings, this study aims to provide robust data support and scientific references for the practice of art education in higher education.

### 3.2 Inclusion and exclusion criteria

**Figure 1:**
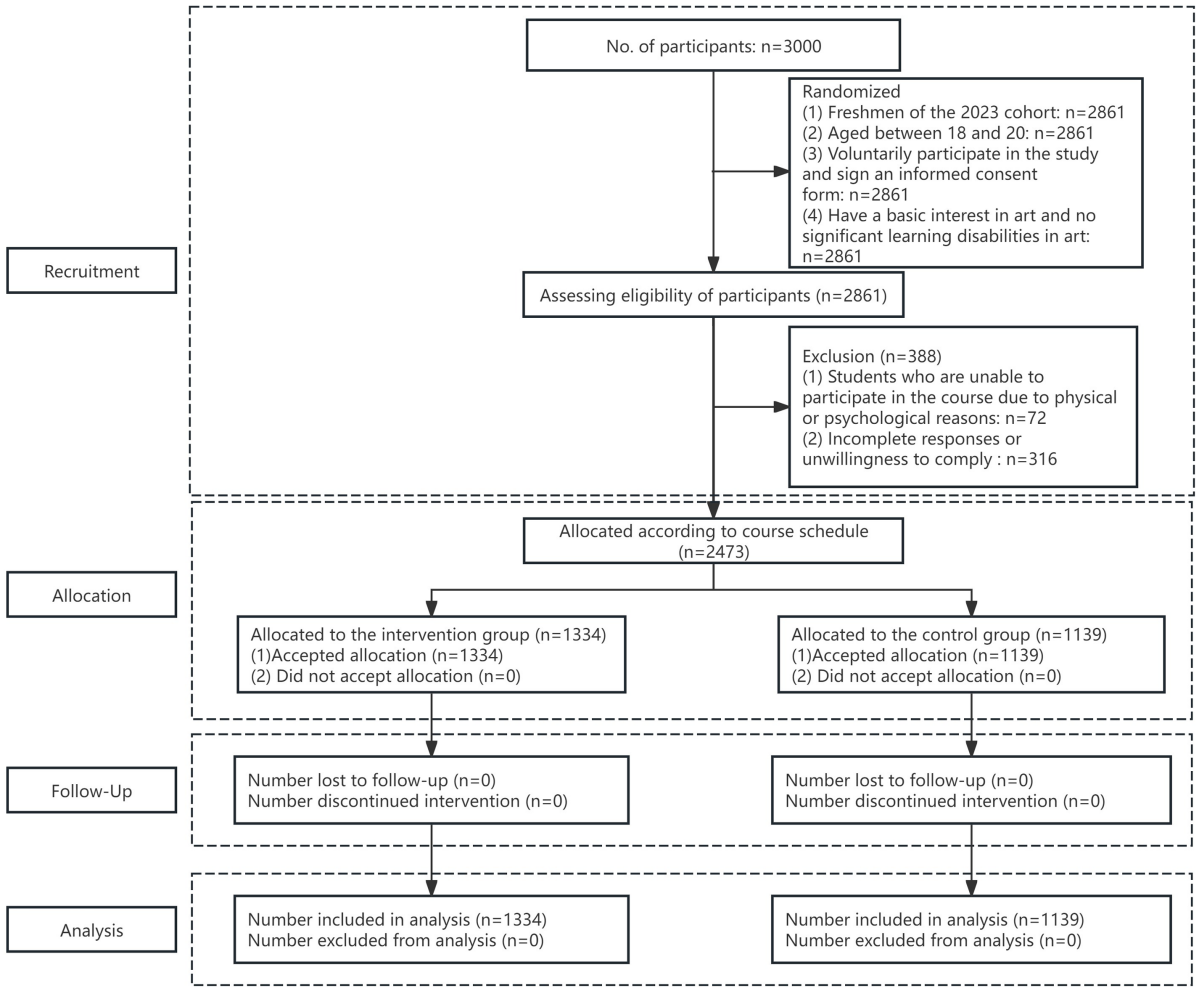
Consort flow diagram.

The CONSORT flowchart of this study is illustrated in Figure 1. The specific inclusion and exclusion criteria are as follows:

Inclusion Criteria:

1. 2023 Undergraduate Freshmen: Must be first-year undergraduate students who enrolled in 2023.
2. Ages between 18 and 22: To ensure homogeneity of the sample, only students within this age range are included.
3. Voluntary participation and signed informed consent: All participants must voluntarily join and sign an informed consent form before the study begins, ensuring they understand the purpose, process, and potential risks of the research.
4. Participants should have a basic interest in art and no significant barriers to learning art: Participants should be interested in art activities and should not have significant learning disabilities related to art, ensuring their adequate participation in this study.

Exclusion Criteria:

1. Students unable to participate in the course due to physical or psychological reasons: This includes, but is not limited to, severe physical illnesses, psychological disorders, or other health issues that may affect the students’ ability to participate adequately in this study.
2. Incomplete responses or unwillingness to comply: Participants who provide incomplete responses or are unwilling to comply with the study requirements will be excluded.

In the specific survey process, this study conducted two tests for two groups of students. The first “pre-test” took place at the end of September 2023, and the second “post-test” was conducted at the end of the course in December 2023 (a 10-week interval between the two measurements). The results of both measurements were matched and summarized using students’ identification numbers. The measurement process involved self-developed questionnaires and the Mental Health Literacy Scale (MHLS) reported by students themselves. The self-developed questionnaire and the MHLS were self-reported by the participants. In the pre-test, 2,861 questionnaires were distributed to 2,861 students. In the post-test, 2,832 questionnaires were distributed to 2,832 students. Due to some students taking leave or dropping out for various reasons, the number of questionnaires distributed in the post-test was inconsistent. After collecting, integrating, and verifying the validity of the data from both distributions, 2,473 valid questionnaires were obtained from 2,473 students, which is more than the required number of valid questionnaires, achieving a recovery and data validity rate of 86.43%. Of these, 1,334 were valid data from students in the experimental group, and 1,139 were from the control group. Additionally, this study has been approved by the university’s ethics committee, and all subjects signed an informed consent form during the questionnaire filling process.

### 3.3 Research tools

#### 3.3.1 Self-developed basic information questionnaire

To comprehensively understand the basic circumstances of the College Students participating in this study and to assess the impact of the public art education courses on their mental health literacy, we designed a detailed basic information questionnaire. The questionnaire covers multiple dimensions to collect students’ personal information and educational background, which are crucial for subsequent data analysis and interpretation. Initially, the questionnaire asks for basic information such as major, age, and gender, which helps us analyze differences between groups. It also examines the students’ province of origin and type of household registration (rural or urban), which can help understand how students’ regional backgrounds might affect their mental health literacy.

Additionally, to explore the potential impact of the family educational environment on students’ mental health literacy, the questionnaire includes an item on the highest educational level of parents. Understanding the educational level of parents helps us analyze the impact of family educational resources on students’ psychological development. Regarding students’ background in art education, the questionnaire asks detailed questions about whether students had studied public art education courses or read related art education books before college. These questions aim to capture the art education experiences and self-study situations of students before formally participating in the public art education offered in this study.

Finally, to assess students’ participation in class, the questionnaire includes an item on the number of times students answered questions during class. This data helps analyze the relationship between students’ classroom activeness and their mental health literacy, exploring whether active participation in class discussions can enhance students’ mental health literacy. The specific reasons are as follows: First, active participation in classroom interactions, especially proactive engagement in public art education courses (such as the frequency of answering questions), is closely related to improvements in mental health literacy. For example, students’ active interactions in class not only enhance their ability to identify psychological disorders but also strengthen their belief in obtaining professional help, promote attitudes conducive to seeking help, and expand their knowledge of mental health information. Second, when students actively answer questions and engage in discussions in class, it not only enhances their understanding and memory of the knowledge but also improves their critical thinking and creative thinking skills. This also aids in their social skills; through interactions with peers and teachers, they learn how to express their own ideas, listen to others, and thus build healthier interpersonal relationships. Third, according to Vygotsky’s sociocultural theory, interaction and social participation are key factors in the development of individual psychological functions. Through classroom interaction and the use of cultural tools, students’ mental health literacy can be effectively promoted. This theory underscores the importance of social interaction in the learning process, indicating that interaction can better facilitate individual development and mental health.

#### 3.3.2 College students’ mental health literacy scale (MHLS)

The mental health literacy scale used in this study was developed by O Connor and Casey (21) and revised by Liu (22). It consists of 23 items covering four dimensions: the ability to recognize psychological disorders (6 items), belief in receiving professional help (7 items), promoting appropriate help-seeking attitudes (6 items), and knowledge of seeking mental health information (4 items). It uses a Likert 5-point scoring method. Higher total scores across these dimensions indicate better mental health literacy. The internal reliability of the mental health literacy scale for College Students, assessed using Cronbach’s alpha, ranged from 0.70 to 0.86, exceeding the acceptable level of 0.60, indicating high reliability across the dimensions. Additionally, validity tests were conducted on the questionnaire data, with a KMO value of 0.8, indicating that the data is suitable for factor analysis. The Bartlett’s Test of Sphericity, if significant at *p* < 0.01, suggests that the data is appropriate for factor analysis.

Additionally, the representation of MHLS in the course is as follows: First, the ability to identify psychological disorders: Public art education courses help students recognize and understand different psychological states by analyzing the emotional and psychological expressions in various artworks. For example, the courses include art creation tasks on mental health themes, where students reflect on and express their own emotions and psychological states during the creation process. Through case study methods combined with actual mental health cases, students’ abilities to identify psychological disorders are further enhanced. Second, the belief in obtaining professional help: The course introduces basic theories and practices of art therapy, showcasing the application and effects of art in psychotherapy. By participating in art projects related to mental health, such as painting therapy or music therapy, students experience the art therapy process firsthand. Through interactive lectures, students’ trust and confidence in professional psychological help are strengthened, encouraging them to seek help when needed. Third, promoting an attitude conducive to seeking help: The course incorporates educational content on mental health assistance, including help-seeking pathways, resources, and methods. Role-playing and simulation exercises train students on how to seek help appropriately in different situations. Group discussions and interactive games help students practice and master help-seeking skills, share and exchange experiences of seeking help, thereby improving their attitudes towards seeking assistance and encouraging them to actively seek help when facing psychological distress. Fourth, acquiring knowledge on seeking mental health information: Public art education courses systematically provide educational content on mental health knowledge, including symptoms, causes, and management of common psychological issues, and recommend and discuss books, articles, and resources related to mental health. Through specialized seminars and the use of multimedia resources, such as documentaries and films, students’ interest and understanding of mental health knowledge are enhanced, expanding their channels and capabilities for accessing mental health information, enabling them to actively learn and apply mental health knowledge.

### 3.4 Research method

Statistical analysis was conducted using SPSS 26.0 software. The total score and scores for each dimension of mental health literacy among college students were all normally distributed. Descriptive statistics were reported as means ± standard deviations. Independent samples t-tests were utilized to compare the MHLS scores of incoming college freshmen under different general circumstances, with subsequent adjustments for multiple comparisons and assumptions of heteroscedasticity.

To ensure the precision and scientific rigor of the statistical analysis in this study, SPSS 26.0 software was chosen, as it is widely employed in the social sciences for statistical analysis. This software enabled detailed statistical analysis of the collected data to ensure the reliability and validity of the results. Prior to data analysis, normality tests were conducted on the total MHLS scores and scores for each dimension among college students. Data that adhere to a normal distribution are more suitable for traditional parametric statistical methods. Thus, after confirming the distribution of all variables and establishing their adherence to a normal distribution, descriptive statistics were presented in the form of means ± standard deviations. This method facilitates a clear representation of the central tendency and dispersion of the data, aiding in subsequent comparative analyses.

To compare the differences in mental health literacy among college freshmen from different backgrounds, genders, and regions, independent samples t-tests were employed to analyze the significance of differences between two groups of data.

The independent samples t-test is particularly useful in comparing average outcomes between groups when the data meet the assumptions of normality and equal variances. However, in cases where these assumptions do not hold, particularly when variances between groups are unequal, the use of Welch’s T-test is recommended. Welch’s T-test is an adaptation of the standard t-test which does not assume equal variances, making it more robust in situations of heteroscedasticity. This test was specifically utilized in this study to provide a more accurate analysis when comparing groups that might not exhibit homogeneity of variance. The inclusion of Welch’s T-test ensures that our findings are more reliable and reflect true differences between groups, irrespective of the underlying distribution of the data. Additionally, to control for the increased risk of Type I errors due to multiple comparisons, adjustments were made. These adjustments are crucial in studies involving multiple independent tests to maintain the integrity of statistical conclusions.

## 4 Research results

### 4.1 Overall distribution of sample data

In this study, 2,473 students participated, divided into 1,334 in the experimental group and 1,139 in the control group. Gender distribution included 1,012 males and 1,461 females, with a higher proportion of females. The majority, 1816, were from urban areas, versus 657 from rural settings, with 1930 from non-coastal and 543 from coastal regions, suggesting a potential geographical bias in university recruitment. Regarding parental education, most families had at least a high school diploma: 34 parents had no formal schooling, 112 had primary, 185 junior high, 655 high school, 1,073 college, and 414 graduate degrees. Prior to college, 1,539 students had not taken public arts courses while 934 had, and about half, 1,206, had read relevant books. Classroom participation was low, with 1,680 students never answering questions, and a smaller number participating one to five times, indicating minimal engagement in class discussions (Table 2).

**Table 2:** Descriptive statistics of basic variables.

Variable name	Group	Number of people
Sample size	Experimental Group	1,334
	Control Group	1,139
Gender	Male	1,012
	Female	1,461
Household registration type	Rural	657
	Urban	1816
Whether the hometown is coastal	No	1930
	Yes	543
Highest education level of parents	Not studying	34
	Elementary School	112
	Middle School	185
	High School	655
	College	1,073
	Graduate School	414
Whether studied public art education course before college	No	1,539
	Yes	934
Whether read relevant books before college	No	1,267
	Yes	1,206
Number of times answered questions in class	0	1,680
	1	344
	2	206
	3	122
	4	77
	5	44

Regarding the distribution of MHLS data, the post-test indicators of mental health literacy among college students were significantly higher than the pre-test indicators. The coefficient of variation (CV) for Pre-test Ability to identify psychological disorders (Pre-test AIPD) was 0.075; Pre-test belief in receiving professional help (Pre-test BRPH) CV was 0.123; Pre-test attitude towards seeking appropriate help (Pre-test ATSAH) CV was 0.111; Pre-test knowledge of seeking mental health information (Pre-test KSMHI) CV was 0.215. The CV for Post-test Ability to identify psychological disorders (Post-test AIPD) was 0.089; Post-test belief in receiving professional help (Post-test BRPH) CV was 0.116; Post-test attitude towards seeking appropriate help (Post-test ATSAH) CV was 0.113; Post-test knowledge of seeking mental health information (Post-test KSMHI) CV was 0.21 (Table 3). Additionally, all major variables followed a normal distribution.

**Table 3:** Descriptive Statistics of MHLS.

Variable name	Sample size	Max	Min	Mean	Standard deviation	Median	Variance	Kurtosis	Skewness	CV	Shapiro–Wilk Test	Kolmogorov–Smirnov Test
Pre-test AIPD	2,473	20	15	17.187	1.282	17	1.643	−0.733	0.085	0.075	0.933 (0.000***)	0.158 (0.000***)
Pre-test BRPH	2,473	25	16	19.679	2.425	20	5.882	−1.048	0.085	0.123	0.946 (0.000***)	0.111 (0.000***)
Pre-test ATSAH	2,473	22	15	17.785	1.978	18	3.914	−0.579	0.4	0.111	0.936 (0.000***)	0.123 (0.000***)
Pre-test KSMHI	2,473	14	5	9.4	2.018	10	4.074	−0.453	−0.389	0.215	0.951 (0.000***)	0.132 (0.000***)
Pre-test MHLS	2,473	79	51	64.051	4.105	64	16.854	0.086	0.149	0.064	0.993 (0.000***)	0.055 (0.000***)
Post-test AIPD	2,473	25	17	20.934	1.866	21	3.483	−0.49	0.316	0.089	0.95 (0.000***)	0.144 (0.000***)
Post-test BRPH	2,473	30	18	23.625	2.731	24	7.461	−0.827	−0.099	0.116	0.968 (0.000***)	0.091 (0.000***)
Post-test ATSAH	2,473	27	16	21.129	2.377	21	5.652	−0.681	−0.067	0.113	0.966 (0.000***)	0.107 (0.000***)
Post-test KSMHI	2,473	20	9	13.571	2.853	13	8.14	−0.912	0.156	0.21	0.959 (0.000***)	0.098 (0.000***)
Post-test MHLS	2,473	98	60	79.26	7.003	80	49.048	−0.636	−0.177	0.088	0.988 (0.000***)	0.067 (0.000***)

***, **, * Represent significance levels of 1, 5, and 10%, respectively.

### 4.2 Differences in pre-test MHLS between urban and rural college students

Regarding the pre-test mental health literacy (MHLS) of college students, urban and rural students showed significant differences in their ability to identify psychological disorders in the pre-test due to heteroscedasticity. Welch’s *T*-test was employed, yielding a significant result with a *p*-value of 0.040**, indicating a significant difference in the ability to identify psychological disorders between urban and rural college students. However, the effect size Cohen’s d was small at 0.105. Similarly, in aspects such as belief in receiving professional help, attitude towards seeking appropriate help, and knowledge of seeking mental health information in the pre-test, Welch’s *T*-tests were used due to heteroscedasticity, and significant statistical results were obtained, indicating significant differences between urban and rural college students in these aspects, although the differences were small. In terms of Pre-test MHLS, as homoscedasticity was met, an independent samples t-test was employed, resulting in a significant *p*-value of 0.000***, indicating a significant difference in Pre-test MHLS between urban and rural areas; with a Cohen’s d value of 0.273, the difference was small (Table 4).

**Table 4:** Results of differences in mental health literacy between urban and rural college students.

Variable name	Variable value	Sample size	Mean	Standard deviation	*T*-test	Welch’s *T*-test	Mean difference	Cohen’s *d* value
Pre-test AIPD	Urban	1816	17.222	1.183	*T* = 2.301 *p* = 0.021**	*T* = 2.052 *p* = 0.040**	0.134	0.105
	Rural	657	17.088	1.518				
	Total	2,473	17.187	1.282				
Pre-test BRPH	Urban	1816	19.893	2.517	*T* = 7.396 *p* = 0.000***	*T* = 8.155 *P* = 0.000***	0.808	0.337
	Rural	657	19.085	2.039				
	Total	2,473	19.679	2.425				
Pre-test ATSAH	Urban	1816	17.574	1.813	*T* = –8.956 *P* = 0.000***	*T* = –8.058 *p* = 0.000***	0.794	0.408
	Rural	657	18.368	2.278				
	Total	2,473	17.785	1.978				
Pre-test KSMHI	Urban	1816	9.656	1.825	*T* = 10.721 *P* = 0.000***	*T* = 9.566 *p* = 0.000***	0.963	0.488
	Rural	657	8.693	2.336				
	Total	2,473	9.4	2.018				
Pre-test MHLS	Urban	1816	64.346	4.048	*T* = 5.988 *P* = 0.000***	*T* = 5.915 *P* = 0.000***	1.112	0.273
	Rural	657	63.234	4.155				
	Total	2,473	64.051	4.105				

***, **, * Represent significance levels of 1, 5, and 10%, respectively.

### 4.3 Differences in pre-test MHLS between different genders of college students

Regarding the pre-test mental health literacy (MHLS) of college students, there were no significant differences between male and female students in their ability to identify psychological disorders, belief in receiving professional help, attitude towards seeking appropriate help, and knowledge of seeking mental health information in the pre-test, as homoscedasticity was met. Independent samples t-tests were employed, resulting in *p*-values greater than 0.05, indicating statistically non-significant results and no significant differences between male and female students in these aspects in the pre-test. However, due to heteroscedasticity, Welch’s T-test was used for Pre-test MHLS, resulting in a non-significant p-value of 0.173, indicating no significant difference between male and female students in Pre-test MHLS (Table 5).

**Table 5:** Differences in mental health literacy between male and female college students.

Variable name	Variable value	Sample size	Mean	Standard deviation	*T*-test	Welch’s *T*-test	Mean difference	Cohen’s *d* value
Pre-test AIPD	Male	1,012	17.187	1.252	*T* = –0.002 *p* = 0.998	*T* = –0.002 *P* = 0.998	0.000	0.000
	Female	1,461	17.187	1.302				
	Total	2,473	17.187	1.282				
Pre-test BRPH	Male	1,012	19.564	2.415	*T* = –1.952 *p* = 0.051*	*T* = -1.954 *P* = 0.051*	0.194	0.08
	Female	1,461	19.758	2.43				
	Total	2,473	19.679	2.425				
Pre-test ATSAH	Male	1,012	17.82	2.029	*T* = 0.73 *p* = 0.466	*T* = 0.724 *p* = 0.469	0.059	0.03
	Female	1,461	17.761	1.943				
	Total	2,473	17.785	1.978				
Pre-test KSMHI	Male	1,012	9.346	2.006	*T* = –1.109 *p* = 0.268	*T* = –1.111 *p* = 0.267	0.091	0.045
	Female	1,461	9.437	2.027				
	Total	2,473	9.4	2.018				
Pre-test MHLS	Male	1,012	63.917	3.95	*T* = –1.347 *p* = 0.178	*T* = –1.362 *p* = 0.173	0.226	0.055
	Female	1,461	64.143	4.209				
	Total	2,473	64.051	4.105				

***, **, * Represent significance levels of 1, 5, and 10%, respectively.

### 4.4 Comparison of pre-test MHLS between experimental and control group college students

To minimize the disparity between the experimental and control groups before the experiment, this study conducted an analysis of pre-test MHLS among students in both groups. The analysis revealed that, before the experiment, there were no statistically significant differences between the experimental and control group students in various dimensions of mental health literacy. This confirmed the initial balance of the experiment. Specifically, there were no statistically significant differences between the experimental and control groups in the pre-test regarding their ability to identify psychological disorders (*p* = 0.485, non-significant, Cohen’s d = 0.028), belief in receiving professional help (*p* = 0.194, non-significant, Cohen’s d = 0.052), attitude towards seeking appropriate help (*p* = 0.163, non-significant, Cohen’s d = 0.056), knowledge of seeking mental health information (*p* = 0.099, non-significant at the 0.05 level, Cohen’s d = 0.067), and pre-test MHLS (*p* = 0.261, non-significant, Cohen’s d = 0.045) (Table 6). This result provided a solid foundation for the study, ensuring that any significant changes in subsequent analyses could be attributed to the experimental intervention itself rather than initial group differences. This rigorous pre-experimental balance test is a crucial step in achieving causal inference, ensuring the reliability and validity of the experimental results.

**Table 6:** Results of differences in mental health literacy between experimental and control group college students at pre-test.

Variable name	Variable value	Sample size	Mean	Standard deviation	*T*-test	Welch’s *T*-test	Mean difference	Cohen’s *d* value
Pre-test AIPD	Experimental Group	1,334	17.17	1.294	*T* = –0.699 *P* = 0.485	T = -0.7 *p* = 0.484	0.036	0.028
	Control Group	1,139	17.206	1.268				
	Total	2,473	17.187	1.282				
Pre-test BRPH	Experimental group	1,334	19.62	2.412	*T* = –1.3 *P* = 0.194	*T* = –1.299 *P* = 0.194	0.127	0.052
	Control group	1,139	19.747	2.44				
	Total	2,473	19.679	2.425				
Pre-test ATSAH	Experimental group	1,334	17.837	2.015	*T* = 1.396 *P* = 0.163	*T* = 1.4 *p* = 0.162	0.112	0.056
	Control group	1,139	17.725	1.934				
	Total	2,473	17.785	1.978				
Pre-test KSMHI	Experimental group	1,334	9.338	2.049	*T* = –1.649 P = 0.099*	*T* = –1.654 *p* = 0.098*	0.134	0.067
	Control group	1,139	9.472	1.981				
	Total	2,473	9.4	2.018				
Pre-test MHLS	Experimental group	1,334	63.965	4.113	*T* = –1.125 *P* = 0.261	*T* = –1.125 *P* = 0.261	0.186	0.045
	Control group	1,139	64.151	4.096				
	Total	2,473	64.051	4.105				

***, **, * Represent significance levels of 1, 5, and 10%, respectively.

### 4.5 Evaluation of the effectiveness of public arts education courses

This study conducted a comparative analysis of mental health literacy (MHLS) among college students in the experimental and control groups after the conclusion of public arts education courses. It was found that, due to heteroscedasticity, Welch’s *T*-test was first employed to compare the abilities of the experimental and control groups in identifying psychological disorders, belief in receiving professional help, attitude towards seeking appropriate help, knowledge of seeking mental health information, and Post-test MHLS. The results of the tests were significant, indicating a significant difference between the experimental and control groups in Post-test MHLS, with a large effect size of Cohen’s *d* value of 2.512 (Table 7).

**Table 7:** Independent sample *T*-test and Welch’s *T*-test results for differences in mental health literacy among college students.

Variable name	Variable value	Sample size	Mean	Standard deviation	*T*-test	Welch’s *T*-test	Mean difference	Cohen’s *d* value
Post-test AIPD	Experimental group	1,334	21.755	1.863	*T* = 26.908 *P* = 0.000***	*T* = 27.598 *P* = 0.000***	1.782	1.086
	Control group	1,139	19.973	1.336				
	Total	2,473	20.934	1.866				
Post-test BRPH	Experimental group	1,334	25.164	2.002	*T* = 38.271 *P* = 0.000***	*T* = 37.805 *P* = 0.000***	3.342	1.544
	Control group	1,139	21.822	2.341				
	Total	2,473	23.625	2.731				
Post-test ATSAH	Experimental Group	1,334	21.993	2.058	*T* = 21.243 *P* = 0.000***	*T* = 21.039 *p* = 0.000***	1.874	0.857
	Control Group	1,139	20.119	2.328				
	Total	2,473	21.129	2.377				
Post-test KSMHI	Experimental Group	1,334	15.404	2.19	*T* = 48.092 *P* = 0.000***	*T* = 48.68 *P* = 0.000***	3.979	1.94
	Control Group	1,139	11.425	1.875				
	Total	2,473	13.571	2.853				
Post-test MHLS	Experimental Group	1,334	84.316	4.191	*T* = 62.254 *P* = 0.000***	*T* = 61.828 *P* = 0.000***	10.978	2.512
	Control Group	1,139	73.338	4.573				
	Total	2,473	79.26	7.003				

***, **, * Represent significance levels of 1, 5, and 10%, respectively.

Furthermore, due to the comparison of multiple groups in the study and the presence of heteroscedasticity, a post-hoc multiple comparison method was adopted to adjust for the potential Type I errors (false positives) that might arise from multiple comparisons. Various methods such as Bonferroni correction were used to ensure the accuracy of the results, which can control the error rate in multiple independent or correlated comparisons and increase the credibility of research findings. The results of the post-hoc multiple comparisons using Bonferroni correction method showed that the mean values for the abilities to identify psychological disorders, belief in receiving professional help, attitude toward seeking appropriate help, knowledge of seeking mental health information, and Post-test MHLS were all ranked higher in the experimental group than in the control group. Significant differences were found between the experimental and control groups. This further demonstrates the robustness of the study results, indicating that public arts education significantly improved the mental health literacy of college students (Table 8).

**Table 8:** *Post hoc* multiple comparison results of differences in mental health literacy among college students.

	(I) Name	(J) Name	(I) Mean	(J) Mean	Difference (I-J)	*P*
Post-test AIPD	Experimental group	Control group	21.755	19.973	1.782	0.000***
Post-test BRPH	Experimental group	Control group	25.164	21.822	3.342	0.000***
Post-test ATSAH	Experimental group	Control group	21.993	20.119	1.874	0.000***
Post-test KSMHI	Experimental group	Control group	15.404	11.425	3.979	0.000***
Post-test MHLS	Experimental group	Control group	84.316	73.338	10.978	0.000***

### 4.6 Does the enthusiasm of college students in class affect the effectiveness of course teaching?

To further investigate the impact of participation in public arts education on students’ mental health literacy, this study conducted regression analysis on data from the experimental group. The results highlighted that active class participation, particularly answering questions, significantly correlates with improved mental health literacy (Table 9). Positive classroom interaction not only enhanced students’ ability to identify psychological disorders but also strengthened their belief in obtaining professional help, fostered appropriate help-seeking attitudes, and broadened their knowledge base about mental health information. Regression analysis also showed a positive link between the frequency of answering questions and overall mental health literacy scores, underscoring that quality and frequency of classroom interaction are crucial for enhancing mental health literacy. Active engagement in discussions and activities boosts students’ understanding, confidence, and self-efficacy, positively affecting various mental health aspects.

**Table 9:** The effect of the number of questions answered by students in public art education courses on mental health literacy.

	(1)	(2)	(3)	(4)	(5)
	Post-test AIPD	Post-test BRPH	Post-test ATSAH	Post-test KSMHI	Post-test MHLS
The number of questions answered in class	0.355^***^	0.728^***^	0.381^***^	0.796^***^	2.261^***^
	(11.43)	(16.49)	(9.52)	(17.65)	(20.83)
Gender	−0.0833	−0.0856	0.0552	0.0178	−0.0960
	(−1.13)	(−0.82)	(0.58)	(0.17)	(−0.37)
Province of origin	−0.0366	−0.195	−0.124	−0.0452	−0.401
	(−0.41)	(−1.55)	(−1.10)	(−0.35)	(−1.30)
Student household registration type	−0.159^*^	−0.608^***^	−0.395^***^	−0.891^***^	−2.053^***^
	(−1.82)	(−4.90)	(−3.51)	(−7.03)	(−6.73)
Highest parental education level	0.187^***^	0.189^***^	0.219^***^	0.243^***^	0.839^***^
	(5.17)	(3.66)	(4.69)	(4.63)	(6.63)
Whether or not public art was studied before college	−0.0806	−0.181	0.00471	−0.212^*^	−0.469^*^
	(−1.04)	(−1.64)	(0.05)	(−1.88)	(−1.73)
Whether or not relevant books were read before college	−0.184^**^	−0.268^**^	−0.128	−0.669^***^	−1.249^***^
	(−2.47)	(−2.53)	(−1.34)	(−6.18)	(−4.80)
_cons	20.41^***^	23.29^***^	20.38^***^	13.22^***^	77.30^***^
	(109.83)	(88.24)	(85.21)	(49.04)	(119.18)
*N*	2,473	2,473	2,473	2,473	2,473
*R*^2^	0.070	0.124	0.050	0.163	0.195
adj. *R*^2^	0.067	0.121	0.047	0.160	0.193

*t* statistics in parentheses. ^*^p < 0.1, ^**^p < 0.05, ^***^p < 0.01.

### 4.7 Will the differences in students’ majors affect the effectiveness of course teaching?

Furthermore, to further analyze the impact of students’ majors on mental health literacy during the public arts education course learning process, this study used multivariate analysis of variance to examine the effect of students’ majors on mental health literacy among students in the experimental group. The study found that based on the grouping variable - major, Wilks’ Lambda’s *p*-value was 0.440, indicating non-significance. Therefore, it was concluded that there were no differences in the overall dependent variable among different groups of majors (Table 10). However, from the specific main effect test results, only students’ majors had a significant impact on the knowledge of seeking mental health information in the Post-test (Table 11).

**Table 10:** Multivariate method analysis test results.

Item	Test method	Statistical value	*F*	*P*
Intercept	Pillai’s Trace	0.998	339	0.000***
	Wilks’ Lambda	0.002	339	0.000***
	Hotelling-Lawley Trace	407.25	339	0.000***
	Roy’s Largest Root	407.25	339	0.000***
Major	Pillai’s Trace	0.044	1,020	0.442
	Wilks’ Lambda	0.956	933.471	0.440
	Hotelling-Lawley Trace	0.045	633.229	0.437
	Roy’s Largest Root	0.035	340	0.036**

**Table 11:** Results of between-subjects effects test.

Item	Dependent variable	Sum of squares	Degrees of freedom	Mean square	*F*	*P*
Intercept	Post-test AIPD	86844.883	1	86844.883	44600.246	0.000***
	Post-test BRPH	104818.397	1	104818.397	25902.307	0.000***
	Post-test ATSAH	73063.606	1	73063.606	57937.353	0.000***
	Post-test KSMHI	28407.117	1	28407.117	14423.952	0.000***
	Post-test MHLS	1117879.414	1	1117879.414	118588.285	0.000***
Major	Post-test AIPD	9.257	3	3.086	1.585	0.193
	Post-test BRPH	3.468	3	1.156	0.286	0.836
	Post-test ATSAH	0.861	3	0.287	0.228	0.877
	Post-test KSMHI	16.706	3	5.569	2.827	0.039**
	Post-test MHLS	54.05	3	18.017	1.911	0.127
Error	Post-test AIPD	665.937	342	1.947		NaN
	Post-test BRPH	1383.965	342	4.047		NaN
	Post-test ATSAH	431.289	342	1.261		NaN
	Post-test KSMHI	673.549	342	1.969		NaN
	Post-test MHLS	3223.883	342	9.427		NaN

## 5 Discussion

This study provided empirical support for the effectiveness of public arts education in enhancing mental health literacy among college students. Specifically, it was observed that students in the experimental group, who participated in public arts education courses, showed significant improvements in their mental health literacy scores from pre-intervention to post-intervention. These improvements spanned several areas, including the ability to identify psychological disorders, belief in receiving professional help, attitudes towards seeking appropriate help, and knowledge in seeking mental health information (58). The control group, in contrast, showed no significant changes, underscoring the potential impact of arts education interventions on student mental health (59). Additionally, the analysis highlighted the critical role of active classroom participation. The positive correlation between classroom interaction and mental health literacy improvements suggests that engagement in course activities can substantially influence the effectiveness of educational interventions. This supports previous findings that suggest engagement and interactivity in learning environments are key factors in educational outcomes (60–62).

Moreover, the regression analysis performed indicated that the frequency of answering questions and engaging in discussions was significantly associated with higher mental health literacy scores. This finding underscores the importance of interactive and participative learning settings in enhancing educational outcomes and mirrors Csikszentmihalyi’s theory of “flow, “where engaging deeply in tasks can significantly alleviate psychological stress and contribute to long-term mental well-being (13, 14).

However, despite the positive findings, some limitations should be acknowledged when discussing their significance. For example, the sample mainly comes from one college, which may limit the generalizability of the research results. Additionally, the study primarily employs quantitative methods, which may not fully capture the deep-seated impacts of arts education on individual psychological changes. Therefore, future research could consider introducing more diverse samples and integrating qualitative methods to delve deeper into the specific mechanisms through which artistic activities influence individual mental health.

Despite the study’s strengths, several limitations warrant consideration: Sample Specificity: The study involved only students from a single university, which may limit the generalizability of the findings to other contexts. Students who volunteer for such studies might not be representative of the general student population, potentially introducing selection bias. Short Duration of the Intervention: The intervention lasted only 4 months. Longer interventions might provide more insight into the sustained impacts of public arts education on mental health literacy. Potential for Selection Bias: The students’ decision to participate might also reflect a pre-existing interest in the arts, which could influence the outcomes. Future research should aim to control for this potential bias to better isolate the effects of the intervention. To address these limitations, future studies should aim to include more diverse and broader samples from multiple institutions. Long-term studies could also help in understanding the persistent effects of public arts education on mental health literacy. Additionally, incorporating a mixed-methods approach might provide deeper insights into the individual psychological changes prompted by arts education, which could be overlooked in purely quantitative assessments.

## 6 Conclusion

This study surveyed 2,473 college students in China, discovering that public arts education significantly enhances their mental health literacy. This finding supports the integration of public arts education into higher education and underscores its role in promoting student well-being. Public arts education was shown to improve students’ knowledge of mental health, emotional management, and psychological coping strategies, primarily due to its positive influence on emotional expression, creativity, and social skills. The research also highlighted the importance of interactive learning environments, as active participation and classroom interaction notably boosted students’ mental health literacy, self-efficacy, and social abilities. Overall, these findings advocate for the expansion of public arts education in higher education to foster students’ mental health and holistic development.

## Data Availability

The original contributions presented in the study are included in the article/supplementary material, further inquiries can be directed to the corresponding author/s.
